# mTOR inhibitor INK128 promotes wound healing by regulating MDSCs

**DOI:** 10.1186/s13287-021-02206-y

**Published:** 2021-03-10

**Authors:** Yi Li, Yujun Xu, Xinhan Liu, Xin Yan, Yue Lin, Qian Tan, Yayi Hou

**Affiliations:** 1 Department of Burns and Plastic Surgery, Nanjing Drum Tower Hospital, The Affiliated Hospital of Nanjing University Medical School, Nanjing, 210093 People’s Republic of China; 2 The State Key Laboratory of Pharmaceutical Biotechnology, Division of Immunology, Medical School, Nanjing University, Nanjing, 210093 People’s Republic of China; 3 Jiangsu Key Laboratory of Molecular Medicine, Nanjing University, Nanjing, 210093 People’s Republic of China

**Keywords:** Myeloid-derived suppressor cells, Immunology, Wound healing, Macrophages

## Abstract

**Background:**

Skin wounds in diabetic patients hardly recover. Accumulating evidence has shown that mammalian target of rapamycin (mTOR) pathway and myeloid-derived suppressor cells (MDSCs) are involved in inflammatory-related response. INK128 is a novel mTOR kinase inhibitor in clinical development. However, the exact roles of MDSCs and INK128 in healing wound of diabetic patients are unclear.

**Methods:**

Mice models of normal, diabetic, and diabetic+INK128 were constructed. Bone marrow (BM)-derived macrophages and RAW264.7 cell line co-cultured with MDSCs, which were induced at different conditions. Flow cytometry, western blot, quantitative real-time PCR, and immunohistochemical analysis were performed.

**Results:**

Diabetic mice (DM) had a slower recovery rate, thinner epidermis and dermis, and less blood vessels than those of normal mice. MDSCs were abnormally accumulated in DM, mTOR was activated in MDSCs of DM, and the cells were treated with high glucose. Moreover, mTOR signaling inhibitor INK128 could promote wound healing through reducing the MDSCs. MDSC function was disordered in DM and high-glucose environments, while INK128 could help retrieve their function. Furthermore, high glucose and other factors in DM could promote M-MDSC differentiation to M1 pro-inflammatory macrophage cells, thus inhibiting wound healing. The differentiation, which was dependent on mTOR signaling, could be reversed by INK128.

**Conclusion:**

INK128 is potential to be developed as a clinical strategy to promote wound healing of diabetic patients.

## Highlights

1. MDSCs regulate wound healing in a mTOR-dependency way.
2. MDSC function was disordered in diabetic mice.
3. M-MDSCs differentiate to M1 macrophages and slow down wound healing.
4. INK128 could promote wound healing of diabetic mice.

## Background

The skin of diabetic patients is prone to damage, prolonged, and unhealed to form refractory ulcers. Diabetic ulcer is one of the common complications of diabetic patients, in which foot ulcerations account for 15% of diabetic patients, and is an important cause of non-traumatic amputation [1], which seriously affects the health and quality of life of diabetic patients. Therefore, finding effective ways to accelerate wound healing in diabetic patients has become an important research direction. Wang et al. [2] reported that hyaluronic acid could be a useful method to heal diabetic wound. Topical simvastatin could accelerate wound healing in diabetic patients by enhancing angiogenesis and lymphangiogenesis [3]. Moreover, the pbFGF-loaded electrospun fibers were reported to might have the function of accelerating skin healing of diabetic patients [4]. Despite this, more therapies were demanded for diabetic patients to promote wound healing.

Wound healing is a multifaceted and dynamic process which includes four overlapping phases of coagulation, inflammation, proliferation, and remodeling, which requires a well-orchestrated integration of the complex biological and molecular events of cells and cell signaling [5]. Diabetic wound healing is characterized by delayed acute and chronic wound unveiling impaired healing due to a postponed, incomplete, or uncoordinated healing process. Especially, diabetic wound exhibits a persistent inflammatory response, making it difficult to transition to the proliferation phase [6]. Myeloid cells, including macrophages and neutrophils, are of vital importance in the process of wound healing mainly by triggering and regulating the inflammatory response [7, 8]. Myeloid-derived suppressor cells (MDSCs) are a group of immature and heterogeneous myeloid cell populations. Increased numbers of MDSCs have been observed under pathological conditions associated with inflammation such as tumors, autoimmune diseases, infection, and obesity [9–13]. MDSCs can be characterized by the expression of CD11b and Gr-1 in mice [14]. It can be further divided into granulocyte-like MDSCs (G-MDSC) and monocyte-like MDSCs (M-MDSCs) [11]. Studies have shown that the proportion of MDSCs in peripheral blood of type I diabetic patients is significantly higher than that of normal people, while the proportion of M-MDSCs decreases [15]. The frequency of CD33^+^HLA^−^DR^−/low^ MDSCs is higher in patients with type 2 diabetic mellitus (DM2) comparing with non-DM2 individuals [16]. Similar conclusion was drawn by Whitfield-Larry et al. [15]; MDSCs are unexpectedly enriched in peripheral blood of both mice and patients with autoimmune diabetes. However, the immunosuppressive function of native T1D MDSCs was impaired. As reported by Shi et al. [14], arctigenin was able to ameliorate inflammation through accumulating G-MDSCs and enhance the immunosuppressive function of MDSCs. This provides us with ideas that the changes both in number and function of MDSCs may be crucial for the development of inflammation state of diabetic mellitus and maladjustment of the wound healing process. Nonetheless, it is still not clear whether accumulation of MDSCs leads to impaired wound healing of diabetes and which factors contribute to the changes of MDSCs in diabetic mellitus.

The mammalian target of rapamycin (mTOR) signaling pathway has been widely recognized to control cell survival, metabolism, and proliferation [17]. It forms the catalytic subunit of two distinct protein complexes, known as mTOR Complex 1 (mTORC1) and 2 (mTORC2) [18]. Studies have suggested that mTOR activity mediates MDSC accumulation in tumor and inflammatory diseases, thereby affecting the outcome of the diseases. Wu et al. reported that the allosteric mTORC1 inhibitor, rapamycin, inhibited MDSC accumulation in tumor and skin allografts [19]. Welte et al. [20] suggested that mTOR signaling in cancer cells dictates a mammary tumor’s ability to stimulate MDSC accumulation through regulating granulocyte-colony-stimulating factor (G-CSF). It has been reported that the second-generation mTOR inhibitor INK128 is an oral, highly effective and selective adenosine triphosphate (ATP) competitor that inhibits mTORC1 and mTORC2 [21, 22]. The first-generation mTOR inhibitor mainly inhibits the complex mTORC1, which may cause negative feedback on the PI3K signaling to be affected, thereby enhancing the phosphorylation activity of AKT and making patients prone to drug resistance. Theoretically, INK128 can overcome the limitation of rapamycin which represents the first generation of mTOR signaling. According to the study of Shi et al. [23, 24], INK128 had a good therapeutic action on lupus and colitis development by regulating MDSCs. The effects of mTOR on diabetes are complex, with the anti- and pro-diabetic effects. Activating of mTOR in β cells can stimulate their proliferation. However, the mTOR activation in immune cells may exacerbate β cell dysfunction, thus aggravating diabetes [25]. Accordingly, we hypothesis that INK128 can affect the course of wound healing in diabetic mice by adjusting MDSCs.

To verify the role of mTOR in MDSCs on regulating the diabetic skin wound healing, we constructed streptozotocin (STZ)-induced diabetic mice and used different concentration of glucose to mimic the MDSC living environment. By focusing on mTOR signaling, we found that a high-glucose environment activated mTOR signaling in MDSCs, which results in aberrant accumulation and differentiation of MDSCs. Moreover, we explored whether in vivo and in vitro treatment with INK128 can accelerate the wound closure of diabetic mice.

## Methods

### Antibodies and reagents

Fluorescein isothiocyanate (FITC)-conjugated anti-mouse CD11b mAb, Allophycocyanin (APC)-conjugated anti-mouse Gr-1 mAb, Allophycocyanin (APC)-conjugated anti-mouse F4/80 mAb, P-phycoerythrin (PE)-conjugated anti-mouse Ly6G mAb, and Allophycocyanin (APC)-conjugated anti-mouse Ly6C mAb were purchased from Biolegend, and β-Tubulin (2144), p-S6 (4858S), S6 (2217S), p-4EBP-1 (2855S), and 4EBP-1 (9644 T) were from cell Signal Technology Inc. mTOR inhibitor INK128 was purchased from Selleckchem. Trizol Reagent and SYBR green dye were bought from Invitrogen. Recombinant mouse IL-6, granulocyte-macrophage colony-stimulating factor (GM-CSF), and MDSC Isolation Kit were obtained from Miltenyi Biotec. Glucose and streptozotocin (STZ) were purchased from Sigma-Aldrich.

### Mice model construction

Male C57/B6 mice (6–8 weeks old) were obtained from the Model Animal Research Center of Nanjing University. They were kept under pathogen-free conditions in 12 h:12 h light and dark cycle. All procedures involving mice were approved by the Medical School for Animal Use and Care Committee of Nanjing University in accordance with guidelines of the US NIH. Diabetic mice were induced by low-dose injections of STZ. Mice were fasted for 5 h and then injected with vehicle or STZ (intraperitoneal (i.p.) injection, 50 mg/kg per day, pH 4, dissolved in 0.1 M sodium citrate buffer) for 5 consecutive days. After blood glucose level keeps steadily over 16.6 mM for 3 weeks, two full-thickness wounds of 5 mm in diameter were made on the dorsal surface of mice. To evaluate the effects of INK128, 1 month after STZ injection, diabetic mice received daily intraperitoneal injection of 1 mg/kg INK128 for another 45 days, then two full-thickness wounds of 5 mm in diameter were made on the dorsal surface. Photos of wound area at 1, 3, 5, 7, 9, and 11 days were taken, and the wound area was quantified by using ImageJ software (National Institutes of Health). Two-millimeter skin region surrounding the wound site was collected at 3 and 7 days for further HE staining, immunostaining, and immunofluorescence. After HE staining, the thicknesses of epidermis and dermis were measured by ImageJ software (National Institutes of Health).

### Generation and isolation of MDSCs

BM cells were isolated from mice by flushing tibiae and femurs as described previously [26]. Spleen-derived MDSCs were purified from control and diabetic mice using Myeloid-Derived Suppressor Cell Isolation Kit. BM cells isolated from mice were cultured in culture medium (sugar-free RPMI 1640 with 10% FBS (Gibco)) supplemented with 40 ng/ml murine IL-6 and 40 ng/ml GM-CSF for 4 days, and added 0, 5, 10, 20, 30, 60, and 120 mM glucose. Moreover, BM cells were supplemented with 0, 25, 50, and 100 nM INK128.

### Macrophage differentiation assay

BM cells were cultured in the presence of 40 ng/ml murine IL-6 and 40 ng/ml GM-CSF and added 5 mM glucose, 30 mM glucose, and 30 mM glucose+ 50 nM INK128 for 4 days. After the different incubation periods, MDSCs were collected and co-cultured with BMDM and RAW264.7 separately. Phenotypes of BMDM and RAW264.7 were determined by flow cytometry analysis.

### Flow cytometry analysis

BM cells, splenocytes, and peripheral blood mononuclear cells (PBMCs) from mice were prepared as single-cell suspensions. To detect mouse MDSC subsets, cells were pre-incubated with FITC-conjugated anti-mouse CD11b mAb and APC-conjugated anti-mouse Gr-1 mAb, then they were stained for 20 min at room temperature in the dark. For the detection of macrophages, cells were labeled with FITC-conjugated anti-mouse CD11b mAb and APC-conjugated anti-mouse F4/80 mAb, and then incubated for 20 min at room temperature in the dark. For the detection of M-MDSC and G-MDSC subsets, cells were labeled with FITC-conjugated anti-mouse CD11b mAb, APC-conjugated anti-mouse Ly6C mAb, and PE-conjugated anti-mouse Ly6G mAb, then cells were incubated for 20 min at room temperature in the dark. Flow cytometry was performed on a FACSCalibur flow cytometer (BD Biosciences). The data was analyzed by the FlowJo software.

### Western blot analysis

The protein expression levels of p-S6, S6, p-4EBP-1, and 4EBP-1 on MDSCs were evaluated. β-Tubulin was used as an internal control in our study. Proteins were extracted on a normal way [27], and the western blot analysis was performed according to Wang et al. [28]. Protein bands were visualized using ECL Plus Western blotting detection reagents (Millipore, Bedford, MA, USA).

### RNA extraction and quantitative real-time PCR

Total RNA of MDSCs were isolated with Trizol Reagent according to the manufacturer’s instructions. Quantitative real-time PCR experiment was performed using SYBR green dye on Step One sequence detection system (Applied Biosystems, Waltham, MA, USA). Relative expression of genes was calculated using 2^−ΔΔCT^ method, with GAPDH as internal control. Primer sequences are as shown in Table 1.

**Table 1:** Primer sequences used in RT-PCR

Gene	Forward primers	Reverse primers
**GAPDH**	AGGTCGGTGTGAACGGATTTG	GGGGTCGTTGATGGCAACA
**p47phox**	ACACCTTCATTCGCCATATTGC	CCTGCCACTTAACCAGGAACA
**gp91phox**	AGTGCGTGTTGCTCGACAA	GCGGTGTGCAGTGCTATCAT
**Arg-1**	CTCCAAGCCAAAGTCCTTAGAG	GGAGCTGTCATTAGGGACATCA
**iNOS**	GTTCTCAGCCCAACAATACAAGA	GTGGACGGGTCGATGTCAC
**IL-6**	CTGCAAGAGACTTCCATCCAG	AGTGGTATAGACAGGTCTGTTGG
**CD206**	CTCTGTTCAGCTATTGGACGC	TGGCACTCCCAAACATAATTTGA
**IL-10**	CTTACTGACTGGCATGAGGATCA	GCAGCTCTAGGAGCATGTGG
**IGF-1**	CACATCATGTCGTCTTCACACC	GGAAGCAACACTCATCCACAATG
**IL-1β**	GAAATGCCACCTTTTGACAGTG	TGGATGCTCTCATCAGGACAG
**S100A8**	AAATCACCATGCCCTCTACAAG	CCCACTTTTATCACCATCGCAA
**S100A9**	GCACAGTTGGCAACCTTTATG	TGATTGTCCTGGTTTGTGTCC

### Histologic and immunohistochemical analyses

Epidermis and dermis and blood vessels of wound skin tissue were obtained from paraffin-embedded tissue, fixed in formalin, and stained with HE, Masson, and CD31, as well as DAPI and CD11b.

### Statistics analysis

Results were expressed as mean ± SEM of three independent experiments and each experiment were tripled. Data between two groups were statistically evaluated by Student’s *t* test. *P* < 0.05 was presented as statistically significant difference.

## Results

### Skin wound recovery of diabetic mice is slow after injury

**Fig. 1:**
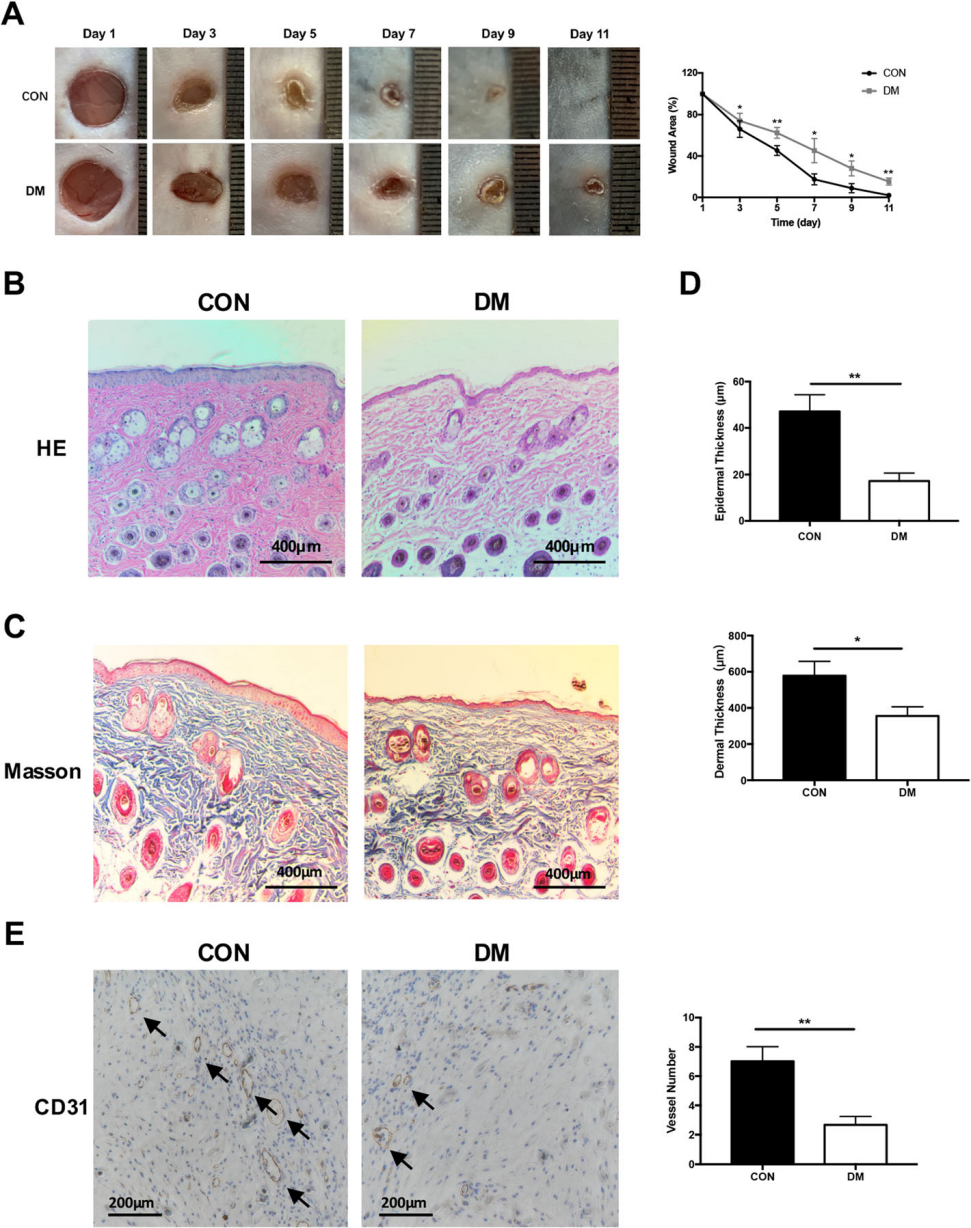
Skin recovery and evaluation of CON and DM after injury. **a** Image of representative wound and area of wound on different days. **b** HE staining of representative wound of CON and DM. **c** Masson staining of representative wound of CON and DM. **d** Evaluation of the thickness of epidermis and dermis in CON and DM. **e** Immunohistochemical staining of CD31 showed the number of endothelium blood vessels of the normal wound and DM wound at 7 days after healing. All data were expressed as the mean ± SEM. *n* = 3, * *P* < 0.05; **P < 0.01. DM, diabetic mice; CON, control mice; HE, hematoxylin eosin

To evaluate the wound closure rate of control mice and diabetic mice, a 5-mm full-thickness round cut on the back was made, which could be normally healed within 11 days. As shown in the images in Fig. 1a, b, the wound closure of diabetic mice was continuously slower than that of control group. The wound area of DM was significantly larger than that of CON on days of 3 (*P* < 0.05), 5 (*P* < 0.01), 7 (*P* < 0.05), 9 (*P* < 0.05), and 11 (*P* < 0.01). On the 11th day of healing, the wound of CON was completely healed, while the DM still had obvious wounds. Then, the thicknesses of epidermis and dermis were assessed. The epidermal thickness of DM exhibited a significant reduction over 60% than CM wound (*P* < 0.01, Fig. 1b, c, d). DM group displayed an ~ 35% reduction in dermal thickness compared with CON group (*P* < 0.05, Fig. 1b, c, d). Moreover, we also calculated the number of endothelium blood vessels in DM and CON. Figure 1e shows that the number of endothelium blood vessels in DM was also significantly less than that of normal mice (*P* < 0.01) at 7 days after injury. Taken together, the DM exhibited a slower recovery rate, thinner epidermis and dermis, and less regeneration of blood vessels than normal mice.

### mTOR inhibitor INK128 promotes skin wound healing and angiogenesis in diabetic mice

**Fig. 2:**
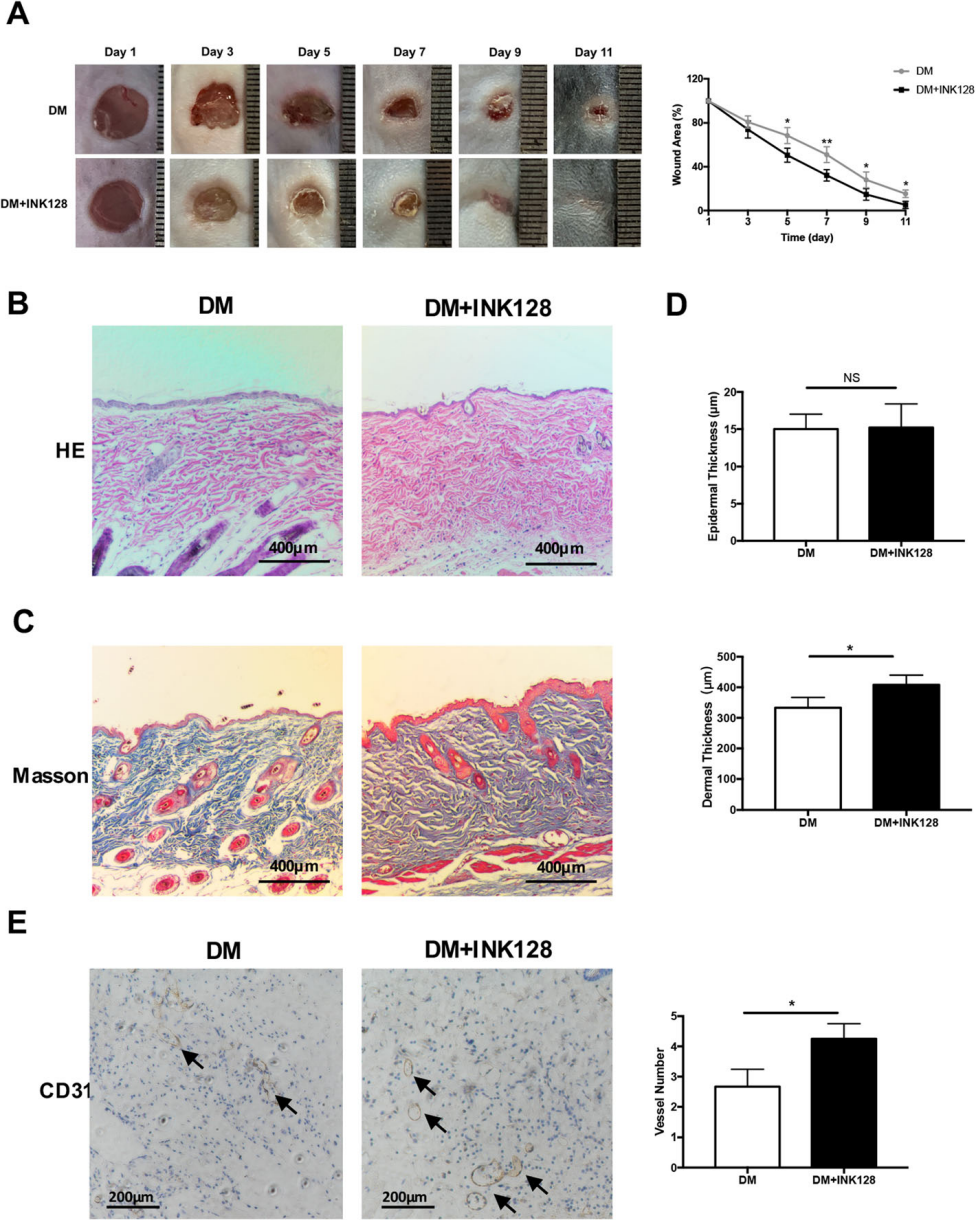
mTOR inhibitor INK128 promotes skin recovery of DM after injury. **a** INK128 decreased the area of wound on different days. **b**–**d** HE staining and Masson staining showed the effect of INK128 on the thickness of epidermis and dermis in DM. **e** Immunohistochemical staining of CD31 showed that INK128 promoted the number of endothelium blood vessels of DM wound at day 7 after injury. All data were expressed as the mean ± SEM. n = 3, * *P* < 0.05; **P < 0.01. DM, diabetic mice; DM + INK128, the diabetic mice treated with INK128

The wound recovery rate of DM and DM + INK128 was assessed. As shown in Fig. 2a, the wound healing rate in DM + INK128 did significantly increased than that in DM. The wound area of DM was significantly larger than that of DM + INK128 on days of 5 (*P* < 0.05), 7 (*P* < 0.01), 9 (*P* < 0.05), and 11 (*P* < 0.05). The epidermal thickness of DM was similar with that of DM + INK128 (*P* > 0.05, Fig. 2b, d), whereas DM displayed a significant reduction in dermal thickness compared to DM + INK128 (*P* < 0.05, Fig. 2b, c, d). Figure 2e shows that the number of endothelium blood vessels in DM was also significantly less than that of DM + INK128 (*P* < 0.01) at day 7, the proliferative phase after injury. These results suggested that INK128 could promote diabetic skin wound healing.

### The percentage of MDSCs is increased in a high-glucose environment

The key for proper wound healing is whether the various reactions in the inflammatory can transition to proliferative phases appropriately [29]. As reported that the MDSCs in peripheral blood mononuclear cell (PBMC) of type I diabetic patients significantly accumulated than in healthy human [15], we doubted that whether the slow recovery of wound was related to the accumulation of MDSCs. Thus, the amount of MDSCs at inflammatory (3 days after injury) and proliferative (7 days after injury) phases in BM, spleen, and PBMC was detected.

**Fig. 3:**
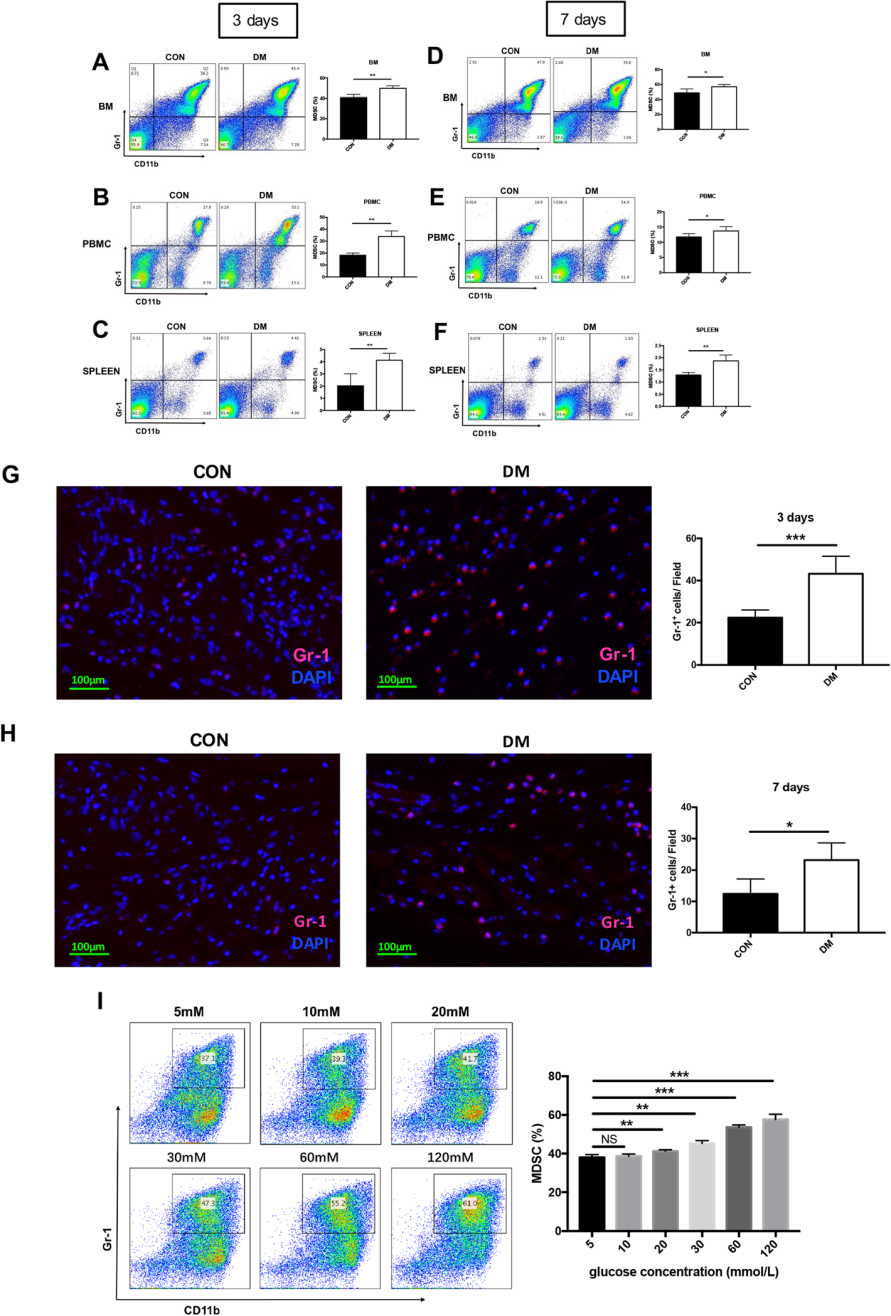
Accumulation of MDSCs in DM and promotion of high glucose on MDSCs. **a**–**c** The accumulation of MDSCs was showed by flow cytometry in bone marrow, PBMC and spleen of DM and CON at day 3 after injury. **d**–**f** The accumulation of MDSCs in bone marrow, PBMC, and spleen at day 7 after injury was showed by flow cytometry. **g**, **h** MDSCs of skin tissue around the wound at day 3 and day 7 after injury were investigated by immunofluorescence staining. **i** The percentage of MDSCs was analyzed by flow cytometry in bone marrow cells from CM treated with the graded concentration of glucose in vitro. MDSCs, myeloid-derived suppressor cells; BM, bone marrow; CON, control mice; DM, diabetic mice; PBMC, peripheral blood mononuclear cell

We found that the percentage of MDSCs in BM, PBMC, and spleen were significantly higher in DM than that in control group both at inflammatory and proliferative phases after injury (Fig. 3a–f; *P* < 0.05). In diabetic mice, the infiltration of MDSCs in the skin tissue around the wound increased on inflammatory and proliferative phases after injury comparing with control mice through immunofluorescence staining Gr-1 of skin tissue (Fig. 3g, h). These results indicate the persistence of inflammation during wound healing process in diabetic mice.

To investigate whether the increased glucose concentration in DM contributed to the accumulation of MDSCs, the BM cells were isolated, treated with IL-6, GM-CSF, and graded glucose of 5, 10, 20, 30, 60, and 120 mM for 4 days to generate MDSCs. Under the gradient addition of glucose in BM cells, the percentage of MDSCs exhibited an increasing trend (Fig. 3i). Then we chose 5 mM and 30 mM glucose concentration for further in vitro experiments corresponding to control mice and diabetic mice, for 25–35 mM is the commonly recognized range in hyperglycemic study. Taken together, the results showed that MDSCs were abnormally accumulated in DM and high glucose promoted the increase of MDSC proportion.

### High glucose promotes the activation of mTOR signaling in MDSCs

**Fig. 4:**
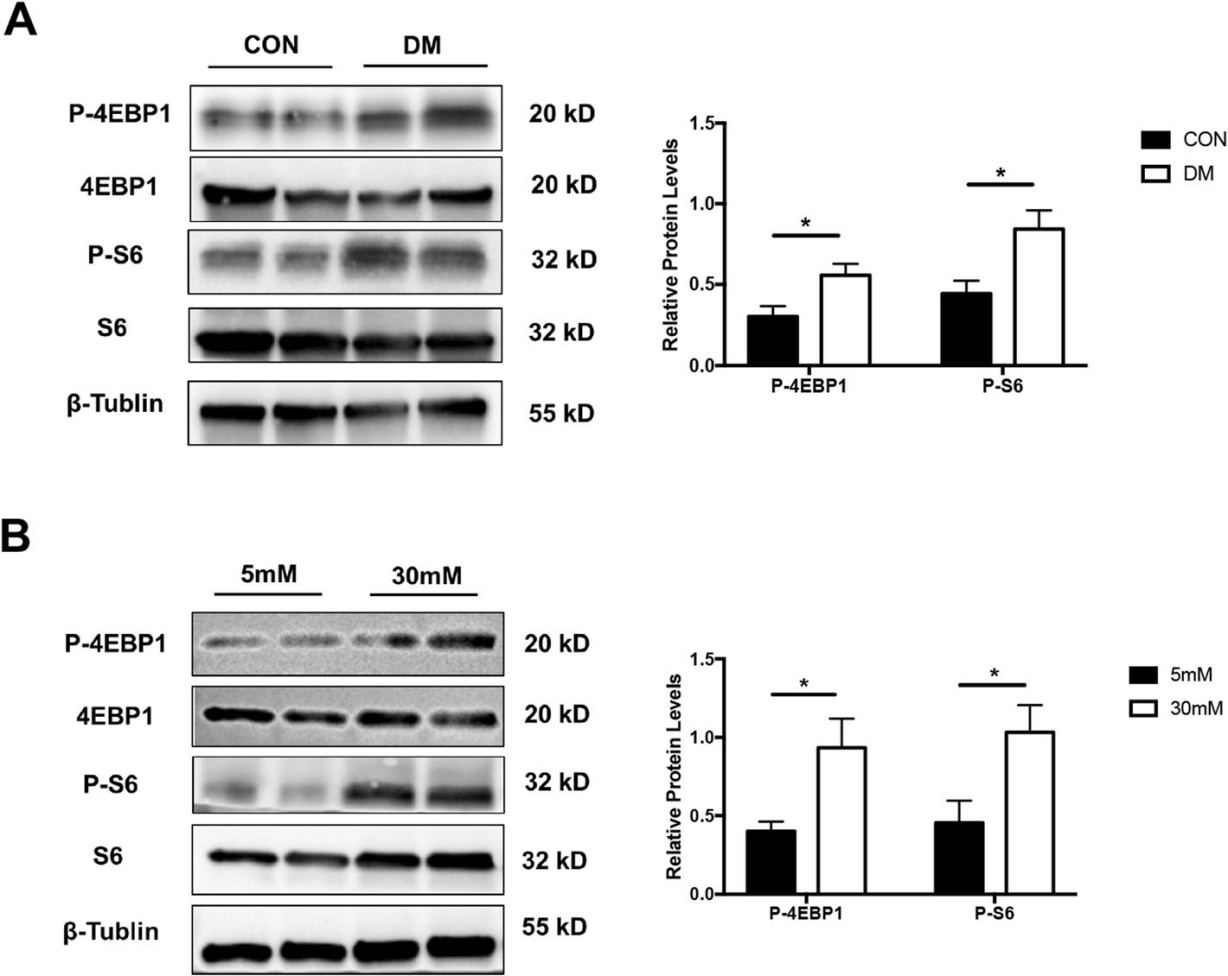
The expressions of the 4EBP1 and S6 along with p-4EBP1 and p-S6 proteins in MDSCs. **a** The expression of 4EBP1, S6, P-4EBP1, and P-S6 was analyzed by Western blot in MDSCs from spleen of CON and DM. **b** The expression of 4EBP1, S6, P-4EBP1, and P-S6 was analyzed by Western blot in MDSCs from bone marrow under presence of 5 mM and 30 mM glucoses, respectively. MDSCs, myeloid-derived suppressor cells; CON, control mice; DM, diabetic mice; P-4EBP1, phosphorylated mammalian target of rapamycin; P-S6, phosphorylated ribosomal protein S6. The experiments were tripled. β-Tublin was used as the reference

To confirm the role of mTOR signaling on accumulation of MDSCs in high glucose, the activation of mTOR pathway was assessed by evaluating the protein expression of downstream signaling molecules phosphorylated mammalian target of rapamycin (p-4EBP1), 4EBP1, and phosphorylated ribosomal protein S6 (p-S6) and S6, with β-Tublin as the reference. Our results showed that p-4EBP1 and p-S6 were significantly highly expressed in MDSCs isolated from diabetic mice than control mice (Fig. 4a). Moreover, the protein expression of p-4EBP1 and p-S6 in BM-derived MDSCs under the presence of 5 mM and 30 mM glucose were also evaluated. The results showed that p-4EBP1 and p-S6 were highly expressed in 30 mM than that in 5 mM (Fig. 4b). These results indicated that mTOR signaling in MDSCs was activated in a high-glucose microenvironment.

### mTOR inhibitor INK128 inhibits the accumulation of MDSCs in high glucose

**Fig. 5:**
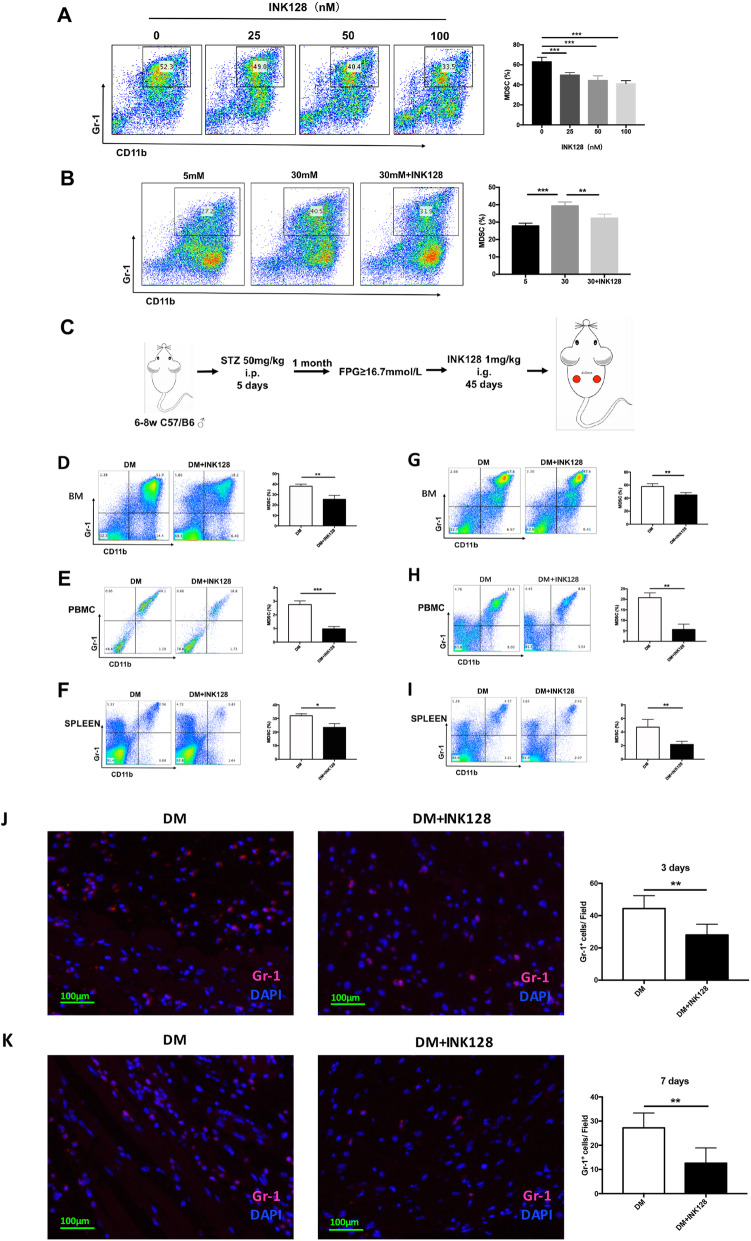
mTOR inhibitor INK128 inhibits accumulation of MDSCs. **a** INK128 inhibited the proliferation of MDSCs in vitro. **b** INK128 decreased the accumulation of glucose-induced MDSCs in vitro. **c** The construction process of DM and DM + INK128 models. **d**–**f** INK128 inhibited the accumulation of MDSCs in marrow, PBMC, and spleen of DM at day 3 after injury. **g**–**i** INK128 inhibited the accumulation of MDSCs in marrow, PBMC, and spleen of DM at day 7 after injury. **j**, **k** Immunofluorescence staining showed that INK128 decreased the accumulation of Gr-1+ cells in skin tissue around the wound of DM at day 3 and day 7 after injury. MDSCs, myeloid-derived suppressor cells; PBMC, peripheral blood mononuclear cell; DM, diabetic mice

To explore the role of mTOR on MDSC expansion, gradient doses (0 nM, 25 nM, 50 nM, and 100 nM) of INK128 were added to BM in the process of generating MDSCs. Figure 5a shows that the percentage of MDSCs under addition of 25 mM, 50 mM, and 100 mM INK128 was significantly decreased compared to control, which demonstrated that INK128 could suppress BM cells that differentiate into MDSCs. BM cells under treatments of 5 mM glucose, 30 mM glucose, and 30 mM glucose + 50 nM INK128 showed that high glucose promoted MDSC expansion, which can be inhibited by INK128 (Fig. 5b).

To further confirm whether INK128 inhibits the accumulation of glucose-induced MDSC expansion in vivo, STZ-induced diabetic mice were treated with vehicle and 1 mg/kg INK128 for 45 days. The percentage of MDSCs in BM, PBMC, and spleen was significantly lower in DM + INK128 group than that in the DM group both at inflammatory and proliferative phases after injury (Fig. 5c–i; *P* < 0.05). In INK128-treated diabetic mice, the infiltration of MDSCs in the skin tissue around the wound significantly decreased at inflammatory and proliferative phases after injury through immunofluorescence staining Gr-1 of skin tissue (Fig. 5j, k). Taken together, these results suggested high glucose caused the accumulation of MDSCs in an mTOR-dependent manner and INK128 inhibited the expansion of MDSCs in vitro and in vivo.

### INK128 suppresses functional gene expression of high-glucose-induced MDSCs

**Fig. 6:**
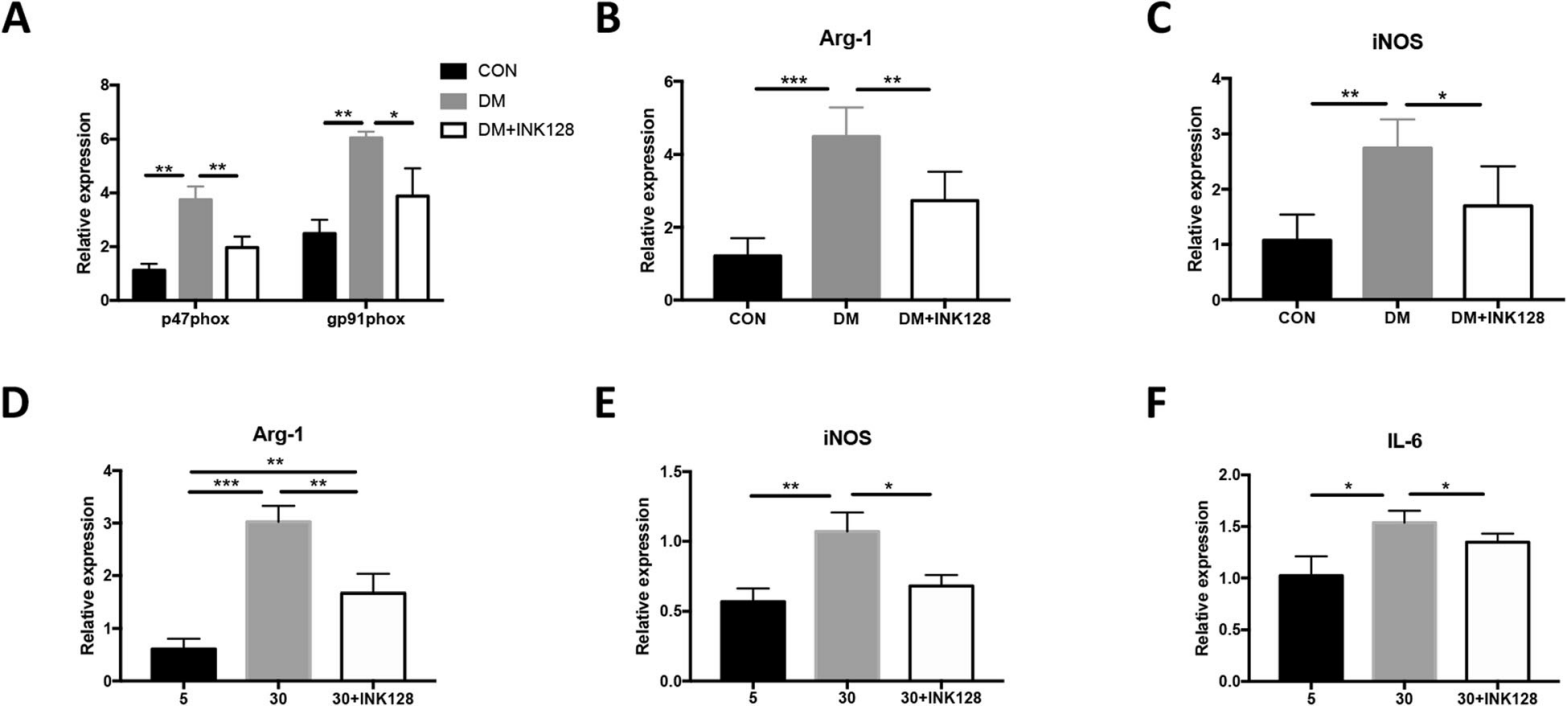
mTOR inhibitor INK128 suppresses the expression of genes related to MDSC functions. **a**–**c** The expressions of p47phox, gp91phox, Arg-1, iNOS, and mRNA in MDSCs purified from the spleen of CON, DM, and DM treated with INK128 by qRT-PCR analysis. **d–f** INK128 decreased the expression of Arg-1, iNOS, and IL-6 in glucose-induced MDSCs in vitro. * *P* < 0.05; **P < 0.01. CON, control mice; DM, diabetic mice

To examine whether the function of MDSC cells was changed in diabetic mice, the MDSCs were isolated from spleens and the expression of several functional molecules including p47phox, gp91phox, arginase-1 (Arg-1), and inducible nitric oxide synthase (iNOS) were detected. The results showed that the expression levels of them were significantly higher in diabetic mice than that in control mice (*P* < 0.05; Fig. 6a–c). Moreover, the effect of INK128 on MDSC function was also evaluated in vitro. The gene expression levels of Arg-1, iNOS, and IL-6 were assessed in MDSCs supplemented with glucose of 5 mM, 30 mM, and 30 mM + INK128. These genes presented a higher expression level in 30 mM glucose than that in 5 mM, and INK128 suppressed the elevation of gene expression in the 30 mM group (*P* < 0.05; Fig. 6d–f). Together, the results demonstrated that the MDSC function was disordered in DM and high-glucose environments, which could explain for the slow wound healing. Moreover, INK128 could help retrieve their function, thus promoting wound healing.

### INK128 inhibits high-glucose-induced differentiation of MDSCs into macrophages

**Fig. 7:**
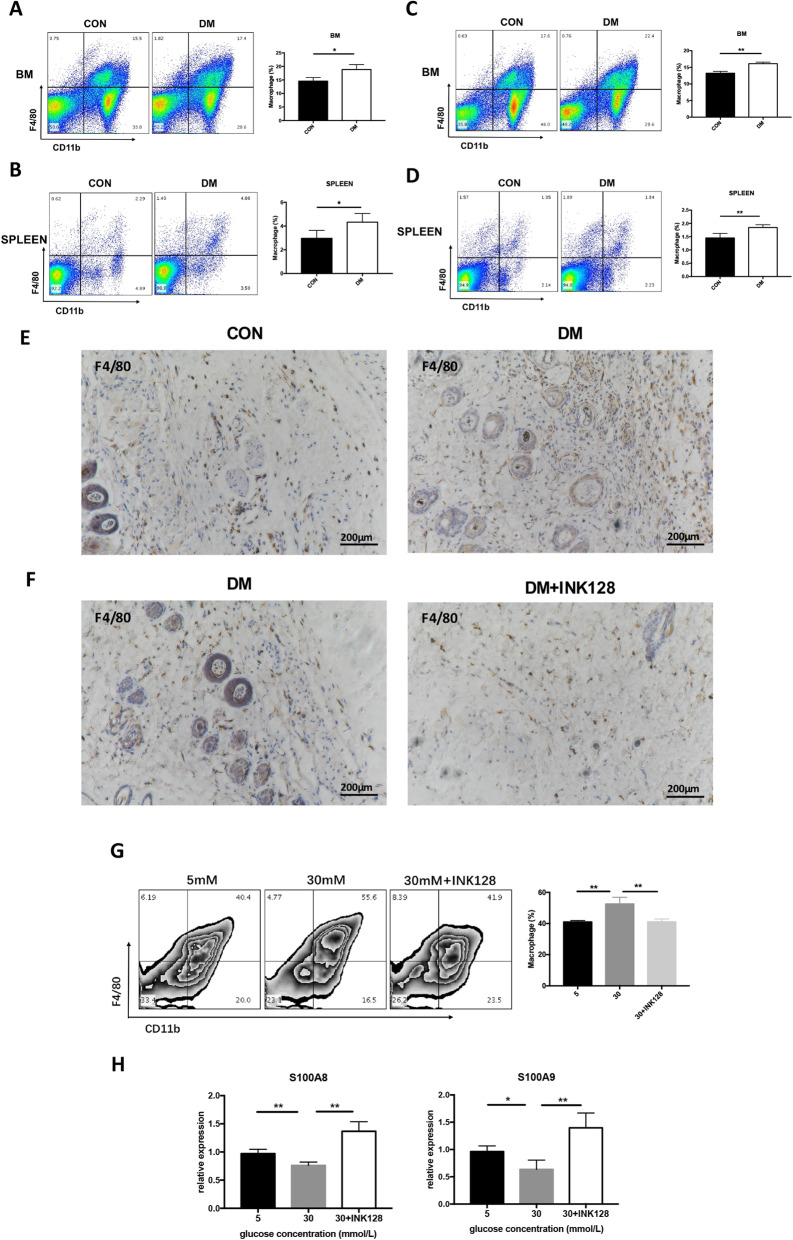
INK128 inhibits differentiation of MDSCs into macrophage in bone marrow and spleen of DM as well as a high-glucose environment. **a**, **b** The percentage of macrophage was increased in bone marrow and spleen of DM. **c**, **d** INK128 decreased the percentage of macrophage in bone marrow and spleen of DM. **e** Immunohistochemistry staining showed that macrophage was increased in skin of DM. **f** INK128 decreased macrophage in skin of DM. **g** the percentage of macrophage under glucose treatments of 5 mM, 30 mM, and 30 mM + INK128 in vitro. **h** INK128 promoted the gene expression of *S100A8* and *S100A9* with glucose treatments. *P < 0.05; **P < 0.01. CON, control mice; DM, diabetic mice; DM + INK128, the diabetic mice treated with INK128

Macrophages are considered as the primary effector cells in regulating wound healing, unregulated macrophage activation represents a source of excessive inflammation, leading to aberrant wound healing [8, 30, 31]. MDSCs have the potential to differentiate to macrophages in chronic inflammation [32]. Diabetes presents a systemic inflammatory state. It is unclear whether high glucose promotes macrophage development and mTOR signaling is involved. Therefore, the amount of macrophage in BM and spleen of CON, DM, and DM + INK128 was detected. Figure 7a–d shows that the percentage of CD11b^+^F4/80^+^ macrophages increased in diabetic mice compared with the control group and INK128 reduced macrophages in DM (*P* < 0.05). Moreover, in the skin tissue around the wound of diabetic mice, massive macrophage infiltration was shown which was mitigated by INK128 treatment (Fig. 7e, f).

To explore whether mTOR signaling was involved in differentiation of MDSCs into macrophages in high glucose in vitro, BM cells were incubated with IL-6 and GM-CSF as well as 5 mM glucose, 30 mM glucose, and 30 mM glucose + INK128 for 4 days. The MDSCs treated with 30 mM glucose displayed a higher macrophage amount than that of 5 mM glucose and 30 mM glucose + INK128 (Fig. 7g), which demonstrated that high glucose could promote MDSCs differentiated into macrophage, in an mTOR-dependent manner. Some studies have demonstrated that S100A8 and S100A9 proteins are directly involved in inhibiting MDSC maturation [32]. Our result showed that INK128 significantly increased the expression levels of S100A8 and S100A9 (*P* < 0.05; Fig. 7h). Taken together, high glucose promoted MDSCs to differentiate into macrophage, and INK128 suppressed the differentiation.

### INK128 reduces M-MDSCs differentiated into pro-inflammatory macrophages induced by high glucose

**Fig. 8:**
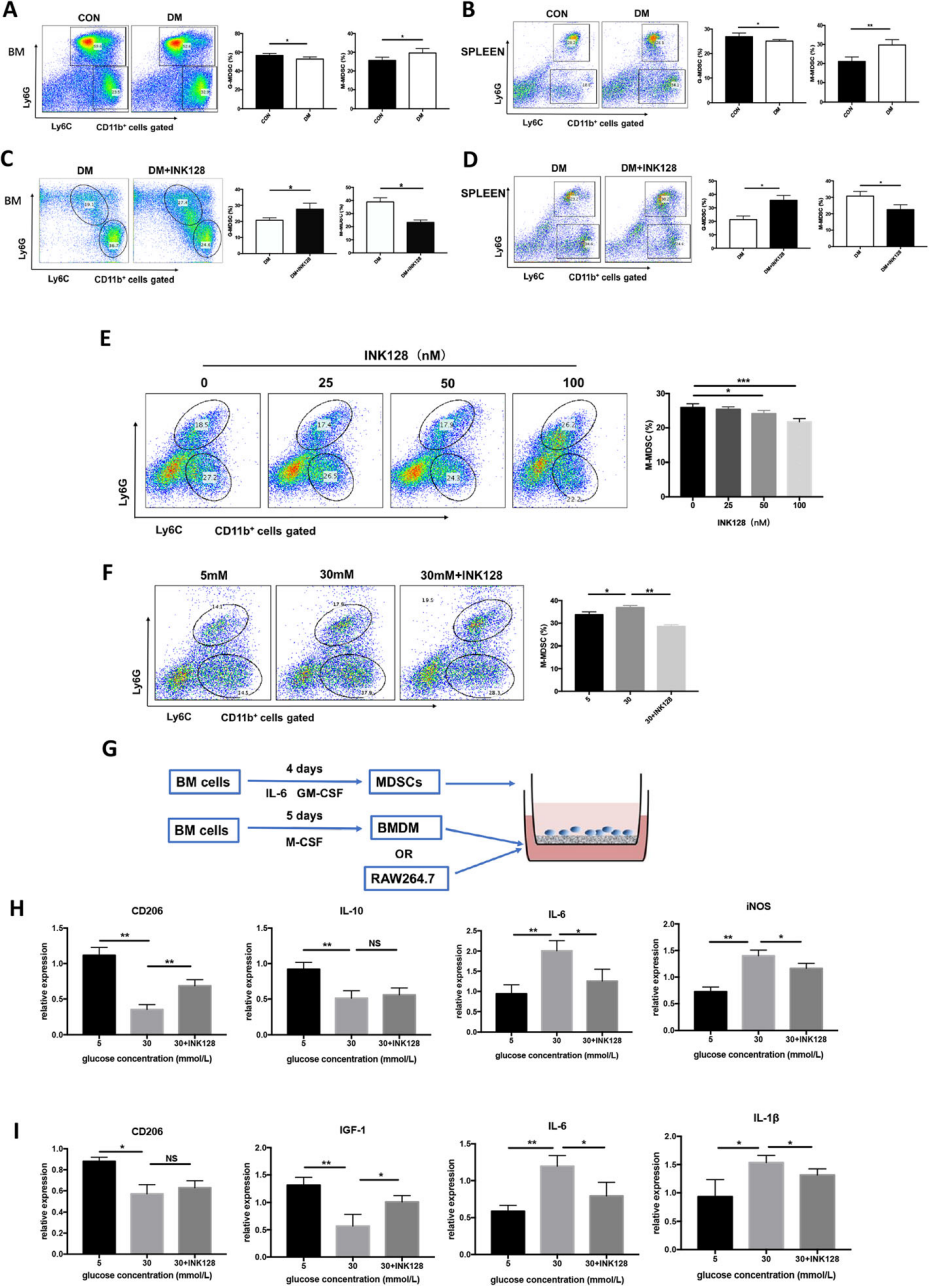
INK128 inhibits the differentiation of M-MDSCs into M1 macrophages in DM. **a**, **b** The MDSCs differentiated into M-MDSCs in bone marrow and spleen of DM. **c**, **d** INK128 decreased the MDSCs to differentiate into M-MDSCs in bone marrow and spleen of DM. **e, f** INK128 inhibited the differentiation of glucose-induced MDSCs into M-MDSCs in vitro. **g** The construction process in differentiation of MDSCs/M-MDSCs into macrophage models. **h, i** INK128 inhibited mainly the differentiation of glucose-induced M-MDSCs into M1 macrophage. * *P* < 0.05; **P < 0.01. M-MDSCs, M-myeloid-derived suppressor cells; CON, control mice; DM, diabetic mice; DM + INK128, the diabetic mice treated with INK128

The phenotype of mice MDSCs is CD11b^+^Gr-1^+^, which can be further divided into two subtypes, including G-MDSCs and M-MDSCs. It was reported that M-MDSCs are the subtype which can differentiate into macrophages; therefore, we detected the percentage of M-MDSCs. Figure 8a–d demonstrates that the percentage of M-MDSCs in DM was significantly higher than that in CON and INK128 reduces the M-MDSC in DM (*P* < 0.05). In vitro, the percentage of M-MDSCs decreased within the increase of INK128 (Fig. 8e). Moreover, the percentage of M-MDSCs in 30 mM glucose group was significantly higher than that in 5 mM glucose and 30 mM glucose + INK128 (*P* < 0.01; Fig. 8f). In summary, the results suggested the percentage of M-MDSCs increased in diabetes and a high-glucose environment, which can be inhibited by INK128.

Macrophages can be divided into pro-inflammatory (M1) and anti-inflammatory (M2) types. In the early stage of wound formation, M1 macrophages infiltrate the periwound tissue, swallow pathogens and necrotic tissue, and play a cleaning role. In the later stage, M2 macrophages cells perform repair functions. The continuous presence of M1 type causes persistence of inflammatory state poor wound healing [33]. In our study, inflammatory cell models BMDM and RAW264.7 were co-cultured with MDSCs pretreated with glucose 5 mM, 30 mM, and 30 mM + INK128 (Fig. 8g). Then, the expression of M1 macrophage markers, i.e., IL-6 and iNOS in BMDM cells, IL-6 and IL-1β in RAW264.7 cells, were detected. The expression of M2 macrophage markers, i.e., CD206 and IL-10 in BMDM cells, CD206 and IGF-1 in RAW264.7 cells, were detected. The result showed that the relative expression levels of CD206 and IL-10 were significantly lower, and IL-6 and iNOS were significantly higher under 30 mM glucose treatment in BMDM than that under 5 mM glucose and 30 mM glucose+INK128 (Fig. 8h). The relative expression levels of CD206 and IGF-1 were significantly lower, and IL-6 and IL-1β were significantly higher under 30 mM glucose treatment in RAW164.7 than that under 5 mM glucose and 30 mM glucose + INK128 (Fig. 8i). These results indicated that MDSCs from a high-glucose environment promoted macrophages to differentiate towards M1 type and INK128 suppressed the effect of high glucose. Taken together, these findings demonstrated that high glucose caused M-MDSCs to differentiate into M1 type which can be inhibited by INK128.

## Discussion

The skin of diabetic patients is easily damaged and difficult to cure, which troubles diabetic patients and affects their lives and health. Zhang et al. [34] reported that MDSCs ameliorated acute kidney injury and the protective effect was enhanced by mTOR signal inhibition. As MDSCs were significantly increased in type I diabetic patients [15, 35], we wonder whether the MDSCs could promote wound recovery of diabetic patients through an mTOR-dependent way. In the present study, we obtained the following conclusions: (1) MDSCs were abnormally accumulated in diabetic mice and a high-glucose environment; (2) mTOR signaling pathway promotes abnormal accumulation of MDSCs; moreover, the mTOR inhibitor INK128 can alleviate wound healing by regulating the accumulation of MDSCs; (3) the dysfunction of MDSCs in diabetic mice and a high-glucose environment leads to difficult wound healing; (4) it was the high-glucose environment that promoted M-MDSCs to differentiate into pro-inflammatory macrophages, resulting in difficult wound healing. In the present study, the therapeutic action of mTOR inhibitor INK128 for healing of diabetic wound was covered for the first time. Moreover, these findings provide important theoretical basis for treating diabetic patients with difficult wound healing.

In the study, we found that the diabetic mice had a slower recovery rate, thinner epidermis and dermis, and less blood vessels than control mice. In the previous studies, the healing methods are mainly around increasing angiogenesis and proliferation of endothelial cells [2, 3]. Meanwhile, in the present study, we investigated the connection of wound healing with MDSCs and tried to find another molecular method to enhance wound recovery. The key for wound healing is whether the various reactions in the inflammatory and proliferative phases can be completed on time and appropriately [29], and the MDSCs were detected to be highly accumulated at inflammatory and proliferative phases in diabetic mice and cells in high glucose. The result was consistent with that in diabetic patients [35]. Moreover, we found that the MDSCs were increased in multiple organs (bone marrow, PBMC, and spleen) of the diabetic mice. Furthermore, we found that the MDSC amount was correlated positively with the supplementary of glucose, which might indicate that high glucose in diabetic mice is the main factor responsible for MDSC accumulation.

The increased mTOR activity is related to insulin resistance, and short-term treatment with rapamycin can lead to an increase of insulin sensitivity, thus ameliorating diabetic mellitus. Therefore, we speculated that mTOR inhibitor treatment could promote diabetic wound healing by regulating MDSCs. However long-term and chronic mTOR inhibition by rapamycin or other rapalogs has been associated with glucose intolerance [36]. Since the drug failure of rapamycin may be due to incomplete mTOR suppression, INK128 was selected in our study for its ability to more effectively inhibit mTORC1 and to inhibit mTORC2 additionally [37]. As expected, the results in our present study confirmed that mTOR signaling could promote the MDSCs. When the mTOR inhibitor INK128 is applied to diabetic mice and cells in high glucose, the amount of MDSCs was reduced and the wound healing rate was improved. We demonstrated that the INK128 promotes wound healing through two ways in diabetic mice. Firstly, the function of MDSCs is disordered in diabetic mellitus. We found that the level of ROS (upregulated with P47 and GP91) produced by MDSCs in diabetic mice was significantly higher. The expression of Arg-1 and iNOS in MDSCs also increased significantly. Moreover, the gene expression levels of Arg-1, iNOS, and IL-6 in cells supplemented with high glucose in vitro were also significantly highly expressed. Under pro-inflammatory conditions, human islets produce and release IL-1, resulting in inhibition of β cell function [38]. Besides, stimulation of mTOR in immune cells, such as NKs and CD8 + T cells, amplifies their functions, potentially exacerbating immune-mediated 훽 cell damage and dysfunction [17, 39]. Elevated number of CD8+ T cells are implicated in the pathogenesis of TIDM. In humans and experimental animals with T2DM, migration and infiltration of pancreatic islets with immune cells, especially macrophages, can be elevated. Secondly, mouse macrophages readily express iNOS in response to LPS and IFN-γ, and for this reason, it is recognized as an M1 macrophage marker in mice [40]. In the present study, the expression of iNOS was significantly higher in high-glucose cells. M1 macrophages express CD86 and produce high levels of ROS and pro-inflammatory cytokines, including IL-6 [41], which was highly expressed in a high-glucose environment. Moreover, the M2 macrophages markers, such as CD206, IL-10, and IGF-1, with their expression significantly decreased in a high-glucose environment [42]. In the wound healing process, M1 pro-inflammatory macrophages were supposed to converse to M2 anti-inflammatory macrophage, while in diabetic mice, their function is improperly regulated and caused a prolonged M1 macrophage presence and inefficient transition to the M2 phenotype, with diabetic mice retaining pro-inflammatory characteristics at day 10 after injury [43]. Notably, as the gene expression in 30 mM + INK128 group have the consistent level with the low-glucose group, we suspected that the INK128 might promote macrophages into M2 pro-inflammatory phenotype; moreover, it might promote the transition from M1 to M2 macrophage, thus promoting wound healing. Moreover, mTOR suppression influenced the differentiation of MDSCs, which further confirmed an mTOR-dependent way of MDSCs to regulate wound healing of diabetic mice. Some studies have shown that S100A8 and S100A9 proteins are directly involved in inhibiting the maturation of MDSCs [23]; thus, our study indicated that mTOR inhibitor INK128 have an obvious effect on suppressing MDSCs to M1 macrophages. Together, the result revealed that injury caused M-MDSC accumulation in diabetic mice, which differentiated in M1 macrophages, thus suppressing wound healing. Conversely, mTOR inhibitor INK128 could decrease the percentage of M-MDSCs, recover the function of MDSCs, and inhibit M-MDSCs differentiated to M1 macrophage thus achieving the purpose of promoting wound healing of diabetic mice.

## Conclusion

In summary, we demonstrated that the number and function of MDSCs in diabetic mice was disorder. MDSCs in high glucose were abnormally accumulated and tend to differentiate into M1 macrophages, thus inhibiting wound healing in an mTOR-dependent way. mTOR inhibitor INK128 could reverse high-glucose-induced changes of MDSCs, thus accelerating the wound healing process. Taken together, these findings highlight that INK128 is a potential therapeutic strategy to promote diabetic wound healing.

## Data Availability

All data generated or analyzed during study are included in the article.

## Abbreviations

MDSCs: Myeloid-derived suppressor cells
M-MDSCs: Monocytic-MDSCs
G-MDSCs: Granulogytic-MDSCs
STZ: Streptozotocin
mTOR: Mammalian target of rapamycin
iNOS: Inducible nitric oxide synthase
IGF-1: Insulin-like growth factor-1
ROS: Reactive oxygen species
BM: Bone marrow
PBMCs: Peripheral blood mononuclear cells
GM-CSF: Granulocyte-macrophage colony-stimulating factor
G-CSF: Granulocyte-colony-stimulating factor
PBS: Phosphate-buffered saline
TNF-α: Tumor necrosis factor-α
TGF-β: Transforming growth factor-β
DCs: Dendritic cells
Th17: T help cell 17
Treg: Regulatory T cells
