# Correction to: mTOR inhibitor INK128 promotes wound healing by regulating MDSCs

**DOI:** 10.1186/s13287-021-02569-2

**Published:** 2021-09-06

**Authors:** Yi Li, Yujun Xu, Xinghan Liu, Xin Yan, Yue Lin, Qian Tan, Yayi Hou

**Affiliations:** 1 Department of Burns and Plastic Surgery, Nanjing Drum Tower Hospital, The Affiliated Hospital of Nanjing University Medical School, Nanjing, 210093 People’s Republic of China; 2 The State Key Laboratory of Pharmaceutical Biotechnology, Division of Immunology, Medical School, Nanjing University, Nanjing, 210093 People’s Republic of China; 3 Jiangsu Key Laboratory of Molecular Medicine, Nanjing University, Nanjing, 210093 People’s Republic of China

## Correction to: Stem Cell Research & Therapy (2021) 12:170 https://doi.org/10.1186/s13287-021-02206-y

The original article [1] mistakenly misspelt co-author Xinghan Liu’s name which has since been corrected.
